# The Chemical and Sensory Impact of Cap Management Techniques, Maceration Length, and Ethanol Level in Syrah Wines from the Central Coast of California

**DOI:** 10.3390/molecules30081694

**Published:** 2025-04-10

**Authors:** Emily S. Stoffel, Sean T. Kuster, L. Federico Casassa

**Affiliations:** 1 Food Science & Nutrition Department, California Polytechnic State University, San Luis Obispo, CA 93407, USA; esstoffe@calpoly.edu; 2 Wine & Viticulture Department, California Polytechnic State University, San Luis Obispo, CA 93407, USA; skuster@calpoly.edu

**Keywords:** cap management, submerged cap, extended maceration, volatile chemistry, Syrah, red wine, Pivot© Profile, expert panel, rapid method

## Abstract

The present study examined the effect of different cap management techniques, maceration length, and ethanol levels through chaptalization on the chemical and sensory composition of Syrah wines from the Edna Valley AVA in California. Punch down wines had significantly higher anthocyanins, tannins, and total polymeric pigments compared to all other treatments. In terms of volatile chemistry, the submerged cap wines tended to have a higher concentration of esters and terpenes compared to the other treatments. Additionally, ethanol levels were more impactful on the chemical composition of the wines. As expected, chaptalized wines had significantly higher ethanol and glucose + fructose levels but also tended to have higher concentrations of esters and terpenes. Sensory evaluation was done through a modification of the Pivot© Profile method using an expert panel of winemakers (n = 15). The results suggested that cap management and the maceration length were more impactful on the sensory profile than the ethanol level whereby each cap management and maceration length treatment had a distinctive profile. As such, the punch down, chaptalized wines showed higher purple hue and color saturation attributes, blueberry orthonasal aromas as well as jammy and black fruit retronasal aromas. Submerged cap wines were associated with significantly meatier orthonasal aromas and reductive retronasal aromas. Extended maceration wines were characterized by more jammy orthonasal aromas and dried fruit retronasal aromas. However, within the extended maceration wines, the effect of chaptalization was apparent whereby the chaptalized wines showed more acetaldehyde aromas while the non-chaptalized wines were characterized by more herbal aromas.

## 1. Introduction

Two important winemaking practices that impact a wine’s chemical and sensory profile are cap management and the maceration length. Cap management is performed during alcoholic fermentation where the resulting cap is consistently integrated into the fermenting juice through punch downs (plunging), submerged cap, pump-overs, or rack-and-returns (délèstage) to aid with phenolic extraction but also to maintain positive aromatic characteristics in the resulting wine [1,2]. The cap refers to the fermentation solids, that is, skins and seeds, that rises to the top of the fermentation vessel from the release of carbon dioxide during alcoholic fermentation [1]. Cap management is critical as previous research has indicated that wines with minimal cap management tend to have increased reductive aromas likely due to the formation of hydrogen sulfide and other sulfur-based compounds from the minimum oxygen exposure during fermentation [3,4]. Experiments regarding cap management in wine have focused on chemical analysis such as phenolics, including color as well as volatile chemistry [2,5]. However, several studies have also included sensory analysis typically using descriptive analysis [6,7] or temporal sensory techniques [8,9,10,11] all of which required a fully trained panel for the evaluation of the wines.

One such study compared the impact of an eight-week extended maceration, submerged cap, and punch downs in California Merlot wine [9]. The phenolic results showed that the punch downs produced wines with significantly lower levels of tannins compared to the other treatments while the submerged cap wines had a significantly higher concentration of anthocyanins (mg/L malividin-3-glucoside). The same study also examined the impact of cap management techniques on the volatile chemistry of wines, reporting that submerged cap wines had significantly lower levels of hexanol while punch down wines had significantly higher levels of hexanol. Also, submerged cap wines had significantly lower concentrations of all the esters measured except for phenylethyl acetate. However, submerged cap wines did have the highest concentration of methionol which is a sulfur-based compound. Descriptive analysis indicated that the punch down treatment also had the lowest intensity score for astringent texture aligning with the phenolic chemistry, but it was not significantly different from the submerged cap treatment. As for the submerged cap treatment, wines were perceived as having higher pepper aromas but otherwise had the lowest intensity scores for red fruit and alcohol aromas which aligned with the volatile chemistry.

Indeed, a submerged cap is a unique cap management system in that a screen device is installed in the fermentation vessel at the beginning of fermentation to prevent the cap from rising and otherwise involves minimal intervention [2]. This method was employed in a two-vintage study of Italian Barbera wines where the resulting phenolic and volatile chemistry were compared to a control or floating cap management treatment which received two pump-overs per day without air [2]. The results indicated that submerged cap wines had significantly higher anthocyanins at pressing compared to the floating cap wines suggesting that submerged cap wines may have more perceived color. Volatile compounds were analyzed through solid-phase extraction (SPE) and the results showed that floating cap wines have significantly higher C6 alcohols, particularly hexanol compared to submerged cap wines. The increase in hexanol and other C6 alcohols was thought to be caused by enzymatic oxidation which could be another indication as to why floating cap wines have less color. However, this study was limited in that there was no sensory analysis conducted on the wines to determine if there was a difference in the perceived color or aroma as hypothesized.

Another study examined the chemical and sensory impact of plunging (punch downs), submerged cap, accentuated cut edges (ACE, a technique based on mechanically breaking grape skins into small fragments), and a combination of ACE and a submerged cap in Pinot noir wines from New Zealand [6]. Phenolic analysis using a modified Somers assay indicated that submerged cap wines had higher tannins (g/L) than wines that had undergone the plunging treatment, 230 days post-inoculation. However, the submerged cap alone had less tannins compared to the treatments that utilized ACE. For anthocyanin concentration (g/L), submerged cap wines had significantly higher levels compared to all other treatments. The potential impact on the volatile chemistry was also examined in this study using solid-phase microextraction (SPME). Submerged cap wines had higher levels of ethyl butanoate and isoamyl acetate compared to wines that had undergone the plunging treatment. Submerged cap wines also had lower levels of hexanol compared to all the other treatments [6] which aligns with the previously mentioned submerged cap studies of Barbera and Merlot [2,9]. As for the sensory impact, descriptive analysis determined that submerged cap wines had a higher red color and astringency but only compared to the control. Additionally, the submerged cap wines (without the additional ACE treatment) had higher aromas of sweaty/cheesy, drain, and pungent aromas compared to all the other treatments. Based on the previously described studies, submerged cap wines have a tendency towards higher anthocyanins [2,6,9] which are correlated with the color but can have more reductive aromas [6,9] compared to punch downs or pump overs.

Once fermentation has completed, an additional winemaking practice is to leave the wine on skins for a period of time; this practice is known as extended maceration [1]. The practice is utilized as it is thought to enhance the color characteristics and stability, as well as mouthfeel through tannin extraction from the extended amount of time that the wine spends in contact with the skins and seeds [1]. Much of the previously reported research has experimented with how long an extended maceration could last before the wine develops negative sensory characteristics. For example, Canadian Shiraz (Syrah) wines were subjected to a three-day extended maceration and compared to wines that underwent a ten-day cold soak prior to fermentation using a paired-comparison sensory test [12]. The results of the sensory test indicated that the extended maceration wines had a higher perceived color than the cold-soak wines. Interestingly phenolic analysis indicated that the extended maceration wines did not have significantly higher total anthocyanins which are typically associated with color. The sensory results also indicated that the extended maceration wines had significantly higher perceived body/finish but more herbaceous aromas, the latter of which typically have negative aromatic connotations.

Another study looked at the effect of a 20-day extended maceration, as well as saignée and water addition in Washington State Merlot [13]. The phenolic analysis results indicated that a 20-day maceration led to higher levels of polymeric pigments (correlated with color stability) and significantly higher tannins compared to all the other treatments [13]. According to the descriptive analysis results, the saignée, extended maceration wines were strongly correlated with the dynamic and drying subqualities of astringency which are typically associated with a higher astringency perception [14]. The higher level of astringency perception was thought to be due to the extraction of seed tannins. However, the perceived color was not evaluated during the sensory analysis. Several studies by the same research group examined the effect of a 30-day extended maceration among other factors in Washington State Cabernet Sauvignon and Merlot wines [15,16,17]. For example, Cabernet Sauvignon wines underwent 30 days of extended maceration and different regulated deficit irrigation protocols [15]. The descriptive analysis results indicated that the extended maceration wines had a significantly higher perceived red and brown color compared to the control. From a taste and mouthfeel perspective, the extended maceration wines had a significantly higher bitterness and astringency perception compared to the control wines [15]. Additional studies of Merlot with 30-day extended maceration periods showed similar sensory results of higher brown hues with increased astringency and bitterness [15,16]. Chemically, the effect of a 30-day extended maceration led to higher polymeric pigments but lower anthocyanins as well as higher tannins including seed tannins [15,16,17]. Based on previous research, both cap management and the maceration length have a distinctive impact on the chemical and sensory character of the wine.

Ethanol is also an important factor affecting the chemical and sensory composition of wine. The ethanol level in wine is influenced by the fruit ripeness which is measured in °Brix, with higher °Brix levels being associated with more alcohol in the resulting wine [18]. In terms of winemaking interventions, the final ethanol can be controlled by adding water to must or juice in order to lower the final ethanol level or by adding grape concentrate or sucrose (chaptalization) to juice or must to increase the ethanol [18,19]. The ethanol level can affect the volatile chemistry of wines as well, which is thought to be due to the level of polarity of the volatile compound being measured or its molecular weight [20,21]. For example, a study that examined monovarietal Malbec wines with two different ranges of ethanol, low (10.0% to 12.0% *v*/*v*) and high (14.5% to 17.2% *v*/*v*) found that the mean area of three of the measured esters decreased significantly with an increasing alcohol level [20]. Ethanol can also mask certain aromatic characteristics, as supported by previous research particularly for fruit-related aromas [20,22]. Within the same study that assessed the effect of ethanol level in Malbec wines, the descriptive analysis results indicated that the increase in ethanol led to a significant decrease in fresh fruit (fruity, strawberry, plum) and cooked fruit aromas [20]. Similarly, the effect of the ethanol level on sensory perception was assessed using commercial single varietal Cabernet Sauvignon wines and Cabernet Sauvignon-based blends. The DA results indicated that as the ethanol level increased, the perception of fresh fruit aromas as well as floral aromas decreased [22]. Additionally, previous research has shown that an increase in ethanol can increase the bitterness, burning sensations, and astringency (in the case of wines with a high tannin concentration) while decreasing the acidity perception [18,22,23,24].

Common to many of the previously mentioned studies that employed sensory analysis was the use of an evaluation method which requires a specially trained panel. However, a newer method, known as Pivot© Profile (PP) was designed for wine evaluation and does not require the use of a trained panel [25]. Instead, it uses an expert panel that can be made up of winemakers, enologists, and/or sommeliers, all of whom are assumed to have an *a priori* understanding of wine’s sensory attributes and winemaking [26]. The method allows for the rapid evaluation of wines as each sample is compared to a control (Pivot©), and the sample wine is categorized as “less than” or “more than” the Pivot© based on free response descriptions [25,27]. Since its introduction, the PP method has been utilized in several wine sensory research studies and has been particularly popular in Australia [28,29,30]. For example, a study found a moderate correlation between the results of the rapid method and descriptive analysis (DA) results [28]. The study examined the relationship between the PP and DA methods and characterized Syrah wines from regions in Australia, New Zealand, and France [28]. Three panels were conducted, one trained DA panel and two PP panels, with one consisting of only sommeliers and the other consisting of winemakers and enologists. RV coefficient analysis indicated a moderate significant relationship between the sommelier (0.69) and winemaker (0.67) PP panels and the trained DA panel. PP was concluded to be useful in identifying the major sensory differences in the wines. The same research group examined the sensory differences between Australian Shiraz wines from six different regions [29]. PP panels were conducted as a way of screening the wines within a region to be selected for a future DA panel. However, the resulting correspondence analysis of the PP studies was able to show subtle differences between the wines within a region. Another study conducted using PP also examined the differences in regionality with Australian Pinot noir wines [30]. As with the previous study, the correspondence analysis from the PP panel was able to group each region based on the sensory characteristics of the wines. The previous studies indicated that PP could produce viable results for wine sensory analysis [28,29,30]. Additionally, a commonality in all these studies was the use of commercial wines. To the authors’ knowledge, there have been no studies that have utilized PP to evaluate the effect of different winemaking techniques on the resulting wines.

While the previous literature has indicated that the PP method can produce viable results, the method does have certain limitations. For example, since the method is free response, the panelists choose their own attributes which then must be interpreted and transformed into binary data prior to analysis. This can prove to be a time-consuming process as several studies collected data on paper [25,28,29]. Also, there can be potential errors in the interpretation of the results, particularly with the wide range of vocabulary used to describe wines [31]. One such study sought to control for this potential error by creating a hybrid of Pivot© Profile and Check-All-That-Apply (CATA), otherwise known as Pivot CATA where the panelists choose pre-selected attributes from two lists which were designated as “less than” or “more than” the Pivot© to evaluate fermented whey beverages [32]. Regarding the logistics, the previous Pivot© Profile testing for wine has been mainly conducted in an open concept or classroom style setting with all wines being presented at once in clear glasses [25,28,29,30], which increases the risk of bias due to color and comparison between samples that are not the Pivot© [33]. An exception was a study conducted on beer where booths and lighting control were utilized but four or five samples were served to a panelist at a time so that the panels could still be influenced by comparing samples that were not the Pivot© [27]. Another similar study conducted panel testing in a controlled environment where the panelist could only compare the Pivot© and the sample to be tested, but this was performed in fermented whey beverages, not wine [32].

As such, the present study had two main aims. The first objective was to evaluate the chemical and sensory impact of different cap management and maceration techniques at different ethanol levels on Syrah wines. The second objective was to provide a blueprint for the modification of the existing Pivot© Profile method for wine sensory analysis. This was accomplished by eliminating the free response aspect and utilizing an attribute list that was established with the panelists prior to evaluation.

## 2. Results

### 2.1. Basic Chemical Composition

A two-way analysis of variance (ANOVA) was conducted on the basic chemistry of the wines (Table 1). Acetaldehyde was the only basic chemical parameter that was not significantly impacted by either the cap management, maceration length, or chaptalization. For the effect of cap management, pH, titratable acidity, and acetic acid were statistically significant. Submerged cap (SubCap) wines had a significantly higher pH (3.28), and titratable acidity (11.00 g/L) compared to the other cap management procedures. Also, SubCap wines had significantly higher levels of acetic acid (0.61 g/L) compared to the punch down (PD) wines but not the extended maceration (EM) wines. The main effect of chaptalization was statistically significant for ethanol, glucose + fructose, lactic acid, and malic acid. As such, chaptalized (Chap) wines had significantly higher ethanol (15.10% *v*/*v*), glucose + fructose (0.24 g/L), and malic acid levels (0.69 g/L) while non-chaptalized (Nat) wines had significantly higher lactic acid levels (0.64 g/L). Finally, all the statistically significant basic chemical parameters had significant first-order interactions except for acetic acid and acetaldehyde.

### 2.2. Phenolic Composition

Table 2 shows a two-way ANOVA on the phenolic composition of the wines. All the phenolic parameters measured were statistically significant for the main effect of cap management but not chaptalization. PD wines had significantly higher anthocyanins (798 mg/L malvidin 3-glucoside (MLV-3G)), total polymeric pigments (TPP) (2.70), tannins (228 mg/L catechin equivalents (CE)), and total phenolics (844 mg/L CE). EM wines tended to have the lowest concentrations of each phenolic parameter measured except for total tannins where the SubCap wines had the lowest concentration of tannins (125 mg/L CE). The main effect of chaptalization was not statistically significant for any of the phenolic parameters, but there were significant first-order interactions for all the compounds except for total phenolics. To further understand the significant interactions between cap management and chaptalization in phenolic compounds, a one-way ANOVA was conducted (Table S1). The only parameter that indicated significant differences based on the chaptalization status of wines within the same cap management style were anthocyanins (Table S1). As such, the punch down, chaptalized (PD_Chap) wines had significantly higher anthocyanin concentrations (848 mg/L MLV-3G) compared to the punch down, natural (PD_Nat) wines (747 mg/L MLV-3G). Otherwise, significant differences among the phenolic compounds were based on the cap management procedure (Table S1).

### 2.3. Volatile Chemistry

A two-way ANOVA shows the relative concentrations (µg/L) of the detected volatile compounds (Table 3). For the main effect of cap management and maceration length, SubCap wines had significantly higher relative concentrations of ethyl octanoate (225 µg/L), ethyl decanoate (9.73 µg/L), and *β*-damascenone (1.34 µg/L) compared to PD wines as well as EM wines (Table 3). Otherwise, the relative concentrations of hexyl acetate, phenylethyl acetate, and nerolidol were not significantly different between PD and SubCap wines (Table 3). Isoamyl acetate (636 µg/L) was the only ester with significantly higher concentrations in PD wines and not SubCap wines. The only significant compounds that had their highest concentration in EM wines were 1-hexanol (3.39 × 10^3^ µg/L) and benzaldehyde (34.4 µg/L) (Table 3). For the main effect of chaptalization, diethyl succinate (420 µg/L), *trans*-farnesol (4.14 µg/L), and phenylethyl alcohol (2.43 × 10^4^ µg/L) concentrations were significantly higher when the wines were chaptalized. In contrast, Nat wines had a significantly higher concentration of ethyl lactate (3.95 × 10^3^ µg/L) (Table 3). It is important to note that all the previously mentioned compounds had statistically significant first-order interactions.

### 2.4. Modified Pivot© Profile

**Figure 1:**
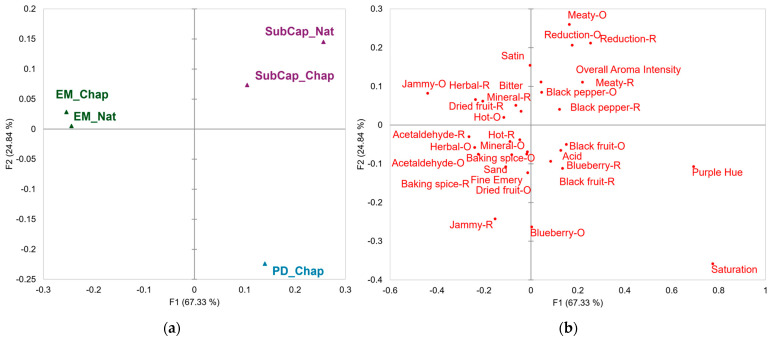
Correspondence analysis showing the relationship between Syrah winemaking treatments and sensory attributes. (**a**) Winemaking treatments. (**b**) Sensory attributes. PD: punch down; SubCap: submerged cap; EM: extended maceration; Nat: natural; Chap: chaptalized; O: orthonasal aroma; R: retronasal aroma.

The results from the modified Pivot© Profile panel are shown in a correspondence analysis (CA), whereby factors one and two explained 92.0% of the variation in the data set (Figure 1). Each cap management treatment, regardless of chaptalization, occupied its own quadrant of the CA, except for the PD_Nat wines which were not characterized, as they were the Pivot© for the present study.

Two color attributes were evaluated by the panel, color saturation and purple hue. Both attributes were associated with PD_Chap wines and inversely related to both EM wines (Figure 1). To further understand the potential significance of sensory attributes in relation to the Pivot©, Fisher’s exact test (*p* < 0.05) was conducted on the color and orthonasal aroma attributes (Table 4) as well as retronasal aromas and mouthfeel attributes (Table 5), but only the statistically significant attributes are shown. According to Fisher’s exact test results, color saturation and purple hue were significant for every treatment except for submerged cap, chaptalized (SubCap_Chap) wines. As such, the PD_Chap wines and submerged cap, natural (SubCap_Nat) wines had significantly higher observed values than expected for purple hue and saturation, meaning that both treatments were cited as “more than” the Pivot© for these attributes (Table 4). Although not statistically significant, the SubCap_Chap wines also tended to have higher observed values for both color saturation and purple hue. In contrast, both EM wines had significantly lower observed color saturation and purple hue in relation to the Pivot© (Table 4).

Thirteen orthonasal aromas with corresponding retronasal aromas were assessed during the sensory evaluation. The CA results showed that the orthonasal aroma attributes tended to appear in the same quadrant as their retronasal aroma counterpart (Figure 1). There were exceptions to this trend, but these descriptors still appeared on the same side of the y-axis to their orthonasal or retronasal aroma counterpart but on different sides of the x-axis. From these attributes, seven orthonasal aromas (Table 4) and eight retronasal aromas (Table 5) were considered statistically significant. The PD_Chap wines were correlated with blueberry and black fruit orthonasal and retronasal aromas compared to the other wines assessed (Figure 1). However, the blueberry orthonasal aroma and the black fruit and jammy retronasal aromas were considered significantly greater in comparison to the Pivot© (Table 4 and Table 5). The PD_Chap wines had significantly less jammy, meaty, and reductive orthonasal aromas compared to the Pivot©.

The SubCap wines were correlated with the overall aroma intensity and the orthonasal–retronasal aroma pairs of black pepper, meaty, and reductive compared to the other treatments (Figure 1). The statistically significant attributes in terms of their relationship to the Pivot© were the meaty orthonasal aroma and reductive retronasal aromas for both SubCap wines (Table 4 and Table 5). Also, the SubCap_Nat wines had a significantly higher overall aroma intensity and meaty retronasal aroma but significantly lower jammy orthonasal and retronasal aromas (Table 4 and Table 5), while the SubCap_Chap wines had significantly lower blueberry orthonasal aromas compared to the Pivot© (Table 4).

As for the EM wines, there was a correlation with the orthonasal and retronasal aromas jammy, acetaldehyde, dried fruit, hot, herbal, mineral, and baking spices (Figure 1). However, EM wines had significantly higher jammy orthonasal aromas and dried fruit retronasal aroma attributes compared to the Pivot© with less meaty retronasal aromas (Table 4 and Table 5). For individual treatments, EM_Nat wines had significantly higher herbal orthonasal and retronasal aroma attributes, while EM_Chap wines had significantly higher acetaldehyde orthonasal and retronasal aroma attributes (Table 4 and Table 5).

For taste and mouthfeel attributes, the correspondence analysis suggested that PD_Chap wines were specifically correlated with acidity, whereas EM wines were associated with the mouthfeel sensation hot (retronasal) and bitterness (Figure 1). However, the only statistically significant attribute in terms of Fisher’s exact test was the subquality of astringency satin. As such, the satin attribute was only significant for the PD_Chap wines meaning that the treatment was cited as having less satin than the Pivot© (Table 5).

## 3. Discussion

### 3.1. Anthocyanin Concentration and Perceived Color

For the main effect of cap management and maceration length, PD wines had significantly higher anthocyanins compared to the SubCap and EM wines, with the EM wines having the lowest concentration of anthocyanins at the time of sensory analysis (Table 2). Anthocyanins are pigments that are correlated with the perception of wine color based on their concentration [34]. There was alignment with the anthocyanin content and the sensory results in the present study based on the correspondence analysis (Figure 1) and Fisher’s exact test (Table 4). Specifically, the PD_Chap and SubCap wines were associated with a greater purple hue and color saturation, while the EM wines were correlated with a lower perceived purple hue and color saturation compared to the Pivot© (PD_Nat).

However, the present results do not align with previous research comparing the impact of punch downs and submerged cap treatments for the anthocyanin content and color perception. Indeed, a study of California Merlot, that was previously mentioned, compared punch downs, submerged cap, and maceration length (0 to 8 weeks), found that submerged cap wines with no extended maceration had higher anthocyanins than the control, but the color was not assessed in the sensory evaluation [9]. Similarly, a study conducted on New Zealand Pinot noir, compared the effect of plunging (punch downs), submerged cap, and ACE. In the said study, the submerged cap wines had significantly higher anthocyanins compared to the other treatments, but the results were based on the Somers assay, which is a different phenolic analysis method than was used in the present study. Nonetheless, the descriptive analysis results indicated that both the submerged cap wines and ACE with submerged cap wines increased the perceived red color [6].

However, the EM anthocyanin concentration and perceived color attribute results of the present study aligned with the previous literature. A previous study that examined the chemical and sensory effects of extended maceration, chaptalization, and harvest time on Washington Merlot over two vintages determined that extended maceration wines had lower anthocyanins compared to the control (punch downs) 120 days post-crushing during both vintages [17]. According to the descriptive analysis results of the study, the extended maceration wines for the 2011 vintage had significantly lower lightness and red color [17]. The loss of anthocyanins and perceived color in the extended maceration wines has been attributed to either the degradation of anthocyanins, the formation of polymeric pigments, or an adsorption of the anthocyanins into the fermentation solids [13,17,35]. In the case of the wines of the present study, EM wines showed a lower color saturation likely either due to a degradation of anthocyanins or adsorption into fermentation solids as the EM wines had significantly lower polymeric pigments compared to the other cap management procedures (Table 2). Regardless of this, the effect of cap management and the maceration length had a distinctive effect on the anthocyanin concentration and perceived color.

The main effect of chaptalization indicated that there was a significant interaction between cap management and chaptalization for anthocyanin concentration (Table 2). The one-way ANOVA results indicated that only the PD wines were significantly different from one another whereby the PD_Chap wines had higher anthocyanins (Table S1). However, the effect of chaptalization was not evident in the sensory evaluation results for color. Due to the nature of the sensory method, the chosen wine treatment for the Pivot© was not characterized during sensory evaluation, and for the present study, the Pivot© was designated as the PD_Nat wines. As such, it is unknown whether there was an alignment between the chemical and sensory data for the impact of chaptalization on anthocyanins and perceived color in the PD wines.

### 3.2. Volatile Chemistry and Aromatic Perception

As for the volatile chemistry, the SubCap wines tended to have either significantly higher ester concentrations (ethyl octanoate and ethyl decanoate), or the relative concentrations (hexyl acetate and phenylethyl acetate) were not significantly different from that of PD wines (Table 3). Esters as well as terpenes can be associated with fruit-related aromas [36,37]. Despite the SubCap wines having higher concentrations of esters and terpenes, the PD wines were sensorially characterized by fruit-driven attributes. Indeed, the PD_Chap wines were correlated with fruit characteristics (blueberry and black fruit), while the SubCap wines were characterized by meaty orthonasal and reductive retronasal aromas compared to the Pivot© (Figure 1).

There are two possible explanations for the lack of alignment between the chemistry and sensory results. First, there could be a masking effect of reductive aromas. Submerged cap management is utilized as a way to limit the oxygen exposure of the cap during fermentation and promote phenolic extraction [2]. However, a limited oxygen exposure, among other factors, can lead to the increased formation of volatile sulfur compounds during fermentation [3,4,38] and thus the potential perception of reduction. For example, the descriptive analysis results from a study that examined the effects of punch downs, submerged cap, and ACE reported that the submerged cap wines had significantly higher pungent, drain, and sweaty/cheesy aromas, all of which are associated with reduction [6]. Similar to the previous study, the submerged cap wines of the present study were associated with greater meaty orthonasal and reductive retronasal aromas. In wines, the meaty attribute can also be associated with volatile sulfur compounds [3]. Previous research examining the sensory effect of volatile sulfur compounds (hydrogen sulfide and methanethiol), on model red wines using Rate-All-That-Apply (RATA) indicated that the presence of reduction-related volatile compounds significantly masked fruit and floral aromas [39], which matches the results herein discussed. However, it is important to note that the volatile chemistry method used in the present study did not include sulfur-based compounds as sulfur-based compounds require a different method and detector for analysis than was available [40]. Another explanation is that the PD_Chap wines could have lacked other defining aromatic characteristics that differentiated the SubCap and even the EM wines. Therefore, it could be suggested that these wines were perceived as more fruit forward compared to the wine that functioned as the Pivot© (Table 5) as well as the other cap management and maceration length treatments. Indeed, the SubCap wines had lower fruit-related orthonasal and retronasal aromas compared to the Pivot© (Table 4 and Table 5).

As for the EM wines, they had significantly greater perceived jammy orthonasal and dried fruit retronasal aromas in comparison to the Pivot© (Table 4 and Table 5). Orthonasal and retronasal dried fruit aromas have been associated with oxidation in red wines [41,42]. The association of both EM wines with dried fruits and jammy orthonasal and retronasal aromas was expected and in agreement with previous research. For example, a study was conducted to examine the chemical and sensory effect of a 30-day extended maceration and different levels of regulated deficit irrigation in Cabernet Sauvignon [15]. The descriptive analysis results indicated that extended maceration wines had a significantly higher oxidative character compared to the control wines which received 10 days of skin contact [15]. Similarly, a separate study of Merlot wines found through descriptive analysis that wines which received punch downs followed by 8 weeks of extended maceration had significantly higher aldehydic aromas compared to all the other treatments including a submerged cap treatment that also received 8 weeks of extended maceration [9].

In the present study, the effect of chaptalization or ethanol level impacted the volatile chemistry mainly through a significant interactive effect between the cap management procedure and chaptalization for both esters and terpenes, whereby chaptalized wines tended to have a higher relative concentration of these compounds. An exception to this trend were ethyl hexanoate and citronellol. The previous literature has indicated that an increase in ethanol level can decrease the concentration of certain compounds, particularly esters [20].

From a sensory perspective, the effect of chaptalization on orthonasal and retronasal aromas was apparent only in the EM wines despite a significant difference in the ethanol level by an average of 2.1% *v*/*v* in all the treatments (Table 1). EM_Nat wines had a significantly greater herbal perception compared to the Pivot© while EM_Chap wines had a significantly greater acetaldehyde perception (Table 4 and Table 5). Syrah wines are known to sometimes display herbal aromas such as mint [43], but otherwise there was not a clear explanation for the attribute’s significant correlation to EM_Nat wines. Interestingly, although EM_Chap wines were associated with acetaldehyde orthonasal and retronasal aromas (Table 4 and Table 5), the acetaldehyde concentration as a factor was not statistically significant for either main effect (Table 1). However, this compound was above the detection threshold in the wines [44]. EM_Chap wines were also associated with the volatile compounds, benzaldehyde and 1-hexanol (Table 3), which have been associated with oxidation-related aromas, but both compounds were below the detection threshold [2,44,45]. Without a clear chemical explanation, the significant correlation of EM_Chap wines to the acetaldehyde orthonasal and retronasal aromas may be due to the comparative sensory nature, where EM_Chap wines were perceived as having more oxidative characteristics in comparison to the Pivot© (PD_Nat wines).

### 3.3. Tannin Concentration and Mouthfeel Perception

Finally, the tannin results indicated that there was a distinctive difference among cap management and maceration length treatments as they were all significantly different from one another. However, the tannin results were considered low for Syrah wines according to previous studies [13,19,46] as there were a range of total tannins among the wines with an average of 112 to 240 mg/L CE (Table 2). This is of note as specific measures were taken to promote phenolic extraction, *i.e.*, tannin concentration such as the fermentation temperature, the selected cap management regimes, and the use of extended maceration in the case of the EM wines [1,2].

Regardless of this, the low levels of tannins aligned with the results obtained by the sensory panel. The tannin content has been associated with the astringency perception in red wines [34]. The sensory results of the present study indicated that there was not a strong association between the wines and the three subqualities of astringency assessed (satin, fine emery, and sand) except for PD_Chap wines and satin (Table 5). Overall, these unusual results may have been due to a vintage effect, or the specific vineyards utilized, but this can only be confirmed by another year of data using the same experimental design and vineyard.

## 4. Materials and Methods

### 4.1. Winemaking

Syrah grapes (clones Alban and 174) of the 2022 vintage were sourced from the Spanish Springs vineyard located in the Edna Valley AVA of the Central Coast of California. The vineyard site is located about three miles away from the Pacific Ocean classifying it as having a cool climate. Fruit (1.50 tons) was harvested in mid-November of 2022 into three, half-ton bins and processed using a destemmer–crusher (Bucher Vaslin, Niederweningen, Switzerland). The two clones were evenly mixed during crushing and subsequently distributed among 106 L stainless steel tank fermenters, so each replicate had 65.0 kg of processed fruit. Winemaking treatments followed a two-by-two full factorial experimental design. The first factor was the cap management technique, which was either in the form of two punch downs daily and was established as the control treatment (PD) or a submerged cap (SubCap). The second factor was the ethanol level: wines labeled as control (Nat) which were fermented without sugar addition and wines labeled as chaptalized (Chap), which were chaptalized with sucrose (First Street, Amerifoods Trading Co., Los Angeles, CA, USA) on the day of fruit processing targeting a potential alcohol level of 15.0% *v*/*v*. For all the treatments, grapes were destemmed and immediately received a 50.0-ppm sulfur dioxide addition at crushing. The must was inoculated with a selected yeast (D20, Enartis, Windsor, CA, USA) at a rate of 30.0 g/hL. All the treatments underwent a five-day cold soak (average temperature of 10.6 °C). An addition of 1.00 g/L of tartaric acid was performed in all the treatments on the first day of the cold soak. After the end of the cold soak, alcoholic fermentation was conducted at an average fermentation temperature of 24.0 °C. Cap management techniques were established on the final day of the cold soak. Submerged cap devices were installed to prevent the cap from rising. Punch downs were performed twice daily (morning and evening) for one minute each until day six of fermentation, and then it was performed for 30.0 sec twice daily until the end of fermentation. Yeast nutrients (Fermaid K, Scott Laboratories, Petaluma, CA, USA) were added at a rate of 30.0 g/hL to the treatments on the last day of the cold soak. All the tanks were inoculated 48.0 h after the initiation of alcoholic fermentation using the malolactic bacteria, *Oenococcus oenii* (VP41, Scott Laboratories, Petaluma, CA, USA).

The wines were fermented to dryness (−1.01 °Brix), and the control (punch downs) and submerged cap treatments were pressed into stainless steel kegs (10.0 L), sparged with Argon and capped tightly with plastic bungs. Half of the control cap management treatments (n = 6) remained in the tanks (106 L) for six months of extended maceration (EM). The full experimental design is shown in Figure S1.

### 4.2. Chemical Analysis

#### 4.2.1. Basic Chemical Composition

The basic chemical compounds were analyzed as previously reported [47]. The pH and titratable acidity were measured manually, the former using a pH meter (Thermo Scientific Orion Star A211, Thermo Fisher Scientific, Waltham, MA, USA) and the latter by titrating a known quantity of wine (5.00 mL) in a deionized water solution against 0.067 N NaOH (Fisher Scientific, Waltham, MA, USA) to a pH endpoint of 8.20. Acetic acid, malic acid, lactic acid, glucose, fructose, and acetaldehyde were measured using commercial enzymatic analysis kits (Admeo, Biosystems Group, Hollister, CA, USA) on an enzymatic analyzer (Admeo Y15, Angwin, CA, USA). Ethanol (% *v*/*v*) was measured via near-infrared spectroscopy using an Alcolyzer Wine M/ME analysis system (Anton Paar, Graz, Austria).

#### 4.2.2. Phenolic Composition

Phenolic compounds were analyzed using spectrophotometric methods on a Cary 60 UV–Vis spectrophotometer (Agilent, Agilent Technologies Inc., Santa Clara, CA, USA) [48,49]. The total phenolics (expressed as mg/L (+)-CE), anthocyanins (expressed as mg/L MLV-3-G), and total polymeric pigments (TPP) [the sum of small polymeric pigments (SPP) and large polymeric pigments (LPP)] were measured using a previously published procedure [48]. Tannins (expressed as mg/L (+)-CE) were analyzed using protein precipitation as previously outlined [49].

#### 4.2.3. Volatile Compound Analysis

Both SPME and stir-bar sorptive extraction (SBSE) methods were utilized for volatile compound analysis. The sample preparation, sampling, and instrument analysis followed a previously outlined procedure [50] with modifications for sampling by SPME fiber [10]. The SBSE method was based on a previously described procedure [51] with modifications for a DB-Wax capillary column [10]. All the volatile compound analysis was carried out on an 8890-gas chromatography system with a 7000D triple quadrupole mass spectrometer detector (Agilent, Agilent Technologies Inc., Santa Clara, CA, USA) as well as an MPS autosampler (Gerstel, Linthicum, MD, USA) using a DB-Wax capillary column, with the dimensions of 30.0 m, 0.250 mm, and 0.250 µm (Agilent, Agilent Technologies Inc., Santa Clara, CA, USA). Helium was used as the carrier gas at a constant flow of 1.33 mL/min [10].

The quantification of the volatile compounds in wine samples was conducted using the internal standard method to calculate the response factor as detailed in a previous procedure [50]. A model wine solution was utilized to optimize the separation of the volatile standards and calculate the response factor for each compound, which was based on a previously described procedure [51] with modifications [10]. The information regarding the volatile compound standards to be quantified including the CAS number, manufacturer, and purity including the internal standard (50.0 µg/L of 2-Undecanone) is provided in Table S2.

### 4.3. Sensory Analysis

#### 4.3.1. Modification to the Pivot© Profile Questionnaire and Selection of Attributes

For the present study, the free response component was replaced by a multiple-choice-style questionnaire (Figure S2) that contained the pre-selected sensory attributes. The questionnaire was created in RedJade sensory software 2023 (RedJade, Pleasant Hill, CA, USA) using a single selection of the grid question type. For each attribute, the group or row was the attribute, and the response or column was defined as “less than the Pivot©” and “more than the Pivot©” which corresponded to the numbers “0” and “1”, respectively, so that each response would automatically be collected in binary.

Attribute and standard references including color, orthonasal aromas, taste, retronasal aromas, and mouthfeel are shown in Table S3. Attributes were selected prior to the panel by a tasting panel of three individuals who were experienced in wine sensory analysis and running trained panels. Even though the attributes were selected prior to the training, the panelists were still allowed to eliminate, add, or modify any attributes before testing if they felt that modifications were needed to better describe the wines.

#### 4.3.2. Panel Training

The project received California Polytechnic State University IRB approval (IRB protocol #2020-058), and all the panelists gave their informed consent prior to their participation in the study. The panelists consisted of winemakers (n = 15) from several wineries within the Central Coast of California. The panel was composed of four females and 11 males ranging in age from 21 to 60 years old. The panelists were screened for both color perception deficiencies and 6-n-propylthiouracil (PROP) sensitivity following previously outlined procedures [10]. None of the panelists displayed color perception deficiencies, and a majority of the panel (80%) were PROP tasters.

The panel training and testing were conducted when the wines had undergone the three-month time point for extended maceration. Both training and testing were performed on one day in February 2023. For training, the panelists were educated on the evaluation procedure, pre-selected standard attributes (Table S3) as well as the research wines. The research wines were presented to the panelists as a blend of all three replicates to help mitigate sensory fatigue, and this included the Pivot© (PD_Nat wines). During the training, the color was assessed separately from the other attributes. This was performed following a previous study in which clear ISO glasses were used to evaluate the color in the wines, and black ISO glasses were used to evaluate the other attributes as the black glasses masked the variation in color among treatments [10]. Once the attributes and standard references were presented to the panel and compared with the tested wines, the panelists were asked whether they wanted to eliminate, add, or modify any of the attributes. For the present study, the panelists did not have any modifications to make to the attribute list. The training in total was a two-hour session, and a fifteen-minute break was given to the panelists prior to the testing.

#### 4.3.3. Panel Testing

Formal evaluations took place in individual sensory booths. The panelists assessed the wines following the evaluation procedures described during the training. The testing was separated by replicate so that each replicate would have two tests, the first assessing the color and the second assessing the orthonasal aroma, taste, retronasal aroma, and mouthfeel. This resulted in six separate tests in total with five wines per test not including the Pivot©. Each replicate had a Pivot©, and the Pivot© remained in the panelist booth during the assessment and was only changed when the panelist completed a replicate, and then a Pivot© of the next testing replicate was provided. The wine was served monadically in 25 mL aliquots at room temperature in clear ISO glasses with 4-digit random codes, and an aluminum foil top. The serving order was randomized according to Williams Latin Square Design.

The color was evaluated under daylight setting lighting (Luna 3, 26:26 W, Zaniboni Lighting, Clearwater, FL, USA) and there was a fifteen-second palate cleansing period between each sample and between replicates. During taste, retronasal aroma, and mouthfeel evaluation, the wines were evaluated under red lighting (Luna 3AO, 18:18 W, Zaniboni Lighting, Clearwater, FL, USA). Additionally, the Pivot© wines were replenished upon request. Following the tasting of each sample, there was a mandatory 30.0 s palate cleansing period where the panelists were provided with unsalted crackers (Nabisco unsalted tops, premium saltine crackers, East Hanover, NJ, USA) and water (Evian natural spring water, Evian, France). All the sensory data collection was conducted in RedJade Sensory Software 2023 (RedJade, Pleasant Hill, CA, USA).

### 4.4. Data Analysis

To understand the relationship between cap management, maceration length, ethanol level, and their interaction in the basic chemistry, volatile chemistry, and phenolic composition data, a two-way ANOVA was conducted (*p* < 0.05) with Fisher’s Least Significant Difference (LSD) as the *post-hoc* comparison. Additionally, a one-way ANOVA was conducted (*p* < 0.05) on the phenolic composition data with Fisher’s LSD as the *post-hoc* comparison.

Due to the binary format of the data, which is like that of CATA data, panel performance was analyzed by using the CATATIS method, specifically examining the agreement among the panelists and the repeatability among the replicates [52]. The results of the panel analysis are shown in Tables S4–S6 which showed strong repeatability and agreement among the panelists. Sensory data preparation and analysis were conducted based on the originally described procedure for the Pivot© Profile which resulted in a CA, with chi square distances, (*p* < 0.05) [25]. Since the binary data were transformed into a contingency table, an additional test known as Fisher’s exact test (*p* < 0.05) was conducted to identify the significant attributes among the wines. Fisher’s exact test determined the direction (lesser or greater) of the observed value in relation to the predicted value of the treatment as it was scored against the Pivot©. All the statistical analyses were run in XLSTAT 2023.3.1 (Lumivero, Denver, CO, USA).

## 5. Conclusions

The present study sought to determine the chemical and sensory impact of cap management, maceration length, and ethanol level in cool-climate Syrah wines. Phenolic results indicated that the cap management style and maceration length had a significant impact on the wines whereby punch down wines had the highest anthocyanin and tannin content compared to the other treatments. However, these findings do not align with the previous literature which has indicated that submerged cap wines tend to have a higher content of anthocyanins. Also, the wines of the present study had an overall low tannin content which is not typical of the Syrah variety. Chaptalization was more impactful on the volatile chemistry, particularly the ester and terpene concentrations. However, this was largely through the significant interactive effects of chaptalization and cap management as opposed to the main effect on its own; nonetheless, chaptalized wines tended to have higher concentrations of these compounds. The lack of alignment of the volatile chemistry with the sensory results was thought to be due to the masking effect of volatile sulfur compounds, or the limitations of the chosen sensory method or a combination of these.

Sensory analysis was conducted using a newly modified version of Pivot© Profile, which was considered successful as there was high panel agreement and repeatability. The results from this analysis indicated that cap management regime and maceration length were more impactful than the effect of chaptalization on the sensory composition of Syrah wines, except for the EM wines. In the case of cool-climate Syrah wines from California, these results suggested that the use of punch downs as a cap management regime could lead to Syrah wines with dark fruit and blueberry characteristics as its defining profile. The submerged cap technique can result in wines with more meaty aromatic characteristics but may be at risk of generating reductive aromas. For submerged cap configurations, we recommend at least two deliberate aeration events throughout fermentation, possibly in the form of a pump-over, to avoid the occurrence of reduction aromas. Additionally, the application of three months of extended maceration resulted in wines with more dried fruit aroma characteristics and less color, but chaptalization led to more oxidized aromas, while no chaptalization (*i.e.*, lower ethanol) led to more herbal aromas. In agreement with previous research, extended maceration tends to result in more “riper” or raisined aromas, whereas conditions conducent to lower ethanol tend to unmask herbaceous or even vegetal aromas. The overall sensory results aligned with the previous literature for the sensory consequences of cap management and Syrah varietal wines in relation to orthonasal and retronasal aromas. While the modification to the Pivot© Profile was considered successful in the present study, future research is required to further validate the adjusted method.

## Figures and Tables

**Table 1:** Two-way analysis of variance (ANOVA) for the factors of cap management, maceration length, chaptalization, and interaction. Values represent the mean of three replicates followed by the standard error of the mean and statistical significance for the basic chemical composition of Syrah wines at the time of sensory evaluation.

Treatment	Ethanol (*v*/*v*%)	pH	Titratable Acidity (g/L)	Glucose + Fructose (g/L)	Lactic Acid (g/L)	Malic Acid (g/L)	Acetic Acid (g/L)	Acetaldehyde (mg/L)
Cap Management								
PD	14.60 ± 0.47	3.11 ± 0.02 b ^1^	11.00 ± 0.16 a	0.17 ± 0.04	0.36 ± 0.11	0.41 ± 0.16	0.50 ± 0.02 b	27.50 ± 3.89
SubCap	13.10 ± 0.50	3.28 ± 0.04 a	10.90 ± 0.22 a	0.17 ± 0.03	0.56 ± 0.11	0.28 ± 0.14	0.61 ± 0.04 a	22.30 ± 3.14
EM	14.40 ± 0.47	3.16 ± 0.01 b	9.56 ± 0.07 b	0.19 ± 0.03	0.41 ± 0.07	0.45 ± 0.16	0.53 ± 0.01 ab	16.70 ± 4.51
*p*-value ^2^	n.s.	**	***	n.s.	n.s	n.s	*	n.s
Chaptalization								
Nat	13.00 ± 0.28 b	3.20 ± 0.25	10.30 ± 0.04	0.11 ± 0.01 b	0.64 ± 0.04 a	0.08 ± 0.01 b	0.54 ± 0.02	24.60 ± 3.31
Chap	15.10 ± 0.24 a	3.16 ± 0.23	10.70 ± 0.02	0.24 ± 0.01 a	0.25 ± 0.05 b	0.69 ± 0.08 a	0.56 ± 0.03	19.70 ± 3.36
*p*-value	***	n.s.	n.s.	***	***	***	n.s.	n.s.
Cap Management × Chaptalization								
*p*-value	**	**	***	**	**	**	n.s.	n.s.

^1^ Different letters within the same column indicate a significant difference for Fisher’s Least Significant Difference test (*p* < 0.05). ^2^ n.s.: not statistically significant; * *p* < 0.05; ** *p* < 0.01; *** *p* < 0.0001. PD: punch down; SubCap: submerged cap; EM: extended maceration; Nat: natural; Chap: chaptalized.

**Table 2:** Two-way analysis of variance (ANOVA) for the factors of cap management, maceration length, chaptalization, and interaction. Values represent the mean of three replicates followed by the standard error of the mean and statistical significance for the phenolic composition of Syrah wines at the time of sensory evaluation.

Treatment	Anthocyanins (mg/L MLV-3G)	SPP	LPP	TPP	Tannins (mg/L CE)	Total Phenolics (mg/L CE)
Cap Management						
PD	798 ± 28.9 a ^1^	1.86 ± 0.05 a	0.84 ± 0.06 a	2.70 ± 0.11 a	228 ± 9.06 a	844 ± 19.9 a
SubCap	715 ± 18.6 b	1.88 ± 0.11 a	0.38 ± 0.08 b	2.26 ± 0.19 b	125 ± 9.16 c	774 ± 17.5 b
EM	519 ± 17.9 c	1.22 ± 0.02 b	0.17 ± 0.04 c	1.39 ± 0.05 c	155 ± 9.02 b	739 ± 18.4 b
*p*-value ^2^	***	***	***	***	***	**
Chaptalization						
Nat	646 ± 41.1	1.61 ± 0.12	0.40 ± 0.10	2.01 ± 0.21	160 ± 16.1	785 ± 21.1
Chap	708 ± 46.1	1.69 ± 0.12	0.53 ± 0.12	2.22 ± 0.22	178 ± 17.0	786 ± 21.6
*p*-value	n.s.	n.s.	n.s.	n.s.	n.s.	n.s.
Cap Management × Chaptalization						
*p*-value	***	**	**	**	***	n.s.

^1^ Different letters within the same column indicate a significant difference for Fisher’s Least Significant Difference test (*p* < 0.05). ^2^ n.s.: not statistically significant; * *p* < 0.05; ** *p* < 0.01; *** *p* < 0.0001. MLV-3G: malvidin-3-glucoside; SPP: small polymeric pigment; LPP: large polymeric pigment; TPP: total polymeric pigment; CE: catechin equivalents. PD: punch down; SubCap: submerged cap; EM: extended maceration; Nat: natural; Chap: chaptalized.

**Table 3:** Two-way analysis of variance (ANOVA) for the factors of cap management, maceration length, chaptalization, and interaction. Values represent the mean of three replicates followed by the standard error of the mean and statistical significance for the relative concentration of detected volatile compounds (µg/L) in Syrah wines at the time of sensory evaluation.

Compounds	Cap Management				Chaptalization			Cap Management × Chaptalization
	PD	SubCap	EM	*p*-value ^2^	Nat	Chap	*p*-value	
Esters								
Ethyl butyrate	129	117	124	n.s.	119	128	n.s.	*
Isoamyl acetate	636 a ^1^	513 b	165 c	***	386	490	n.s.	***
Ethyl hexanoate	494	505	476	n.s.	485	498	n.s.	n.s.
Ethyl lactate	2.89 × 10^3^	2.06 × 10^3^	1.28 × 10^3^	n.s.	3.95 × 10^3^ a	199 b	***	***
Hexyl acetate	6.01 a	5.06 a	n.d. ^3^	**	2.35	5.03	n.s.	**
Ethyl octanoate	167 b	225 a	117 c	**	159	181	n.s.	*
Ethyl hexadecanoate	n.d.	108 a	96.0 a	n.s.	60.5	75.2	n.s.	*
Ethyl decanoate	6.80 b	9.73 a	2.79 c	**	4.94	7.94	n.s.	***
Diethyl succinate	367	336	430	n.s.	336 b	420 a	**	**
Phenylethyl acetate	32.4 a	40.5 a	4.87 b	**	16.7	35.1	n.s.	**
Nor-isoprenoids								
*β*-Damascenone	1.22 b	1.34 a	n.d.	***	0.842	0.860	n.s.	***
Terpenes								
Citronellol	6.73	8.96	9.58	n.s.	9.23	7.62	n.s.	n.s.
*trans*-Farnesol	3.18	3.58	3.02	n.s.	2.38 b	4.14 a	**	*
Nerolidol	7.06 a	7.99 a	1.86 b	***	4.47	6.80	n.s.	***
Alcohols								
1-Hexanol	3.06 × 10^3^ ab	2.68 × 10^3^ b	3.39 × 10^3^ a	**	3.11 × 10^3^	3.00 × 10^4^	n.s.	**
1-Octanol	1.09	n.d.	2.72	n.s.	n.d.	2.54 a	*	**
1-Nonanol	3.44 a	3.06 ab	2.72 b	*	2.77 b	3.37 a	**	**
Isobutanol	1.30 × 10^3^ a	713 b	855 b	*	1.05 × 10^3^	858	n.s.	n.s.
Isoamyl alcohol	1.30 × 10^4^	1.00 × 10^4^	1.03 × 10^4^	n.s.	9.93 × 10^3^	1.23 × 10^4^	n.s.	n.s.
Phenylethyl alcohol	2.23 × 10^4^	2.27 × 10^4^	2.25 × 10^4^	n.s.	2.08 × 10^4^ b	2.43 × 10^4^ a	*	n.s.
Aldehydes								
Benzaldehyde	n.d.	n.d.	34.4 a	***	8.16	14.8	n.s.	**

^1^ Different letters within the same column indicate a significant difference for Fisher’s Least Significant Difference test (*p* < 0.05). ^2^ n.s.: not statistically significant; * *p* < 0.05; ** *p* < 0.01; *** *p* < 0.0001. ^3^ Volatile compound not detected by the analytical method. PD: punch down; SubCap: submerged cap; EM: extended maceration; Nat: natural; Chap: chaptalized.

**Table 4:** Fisher’s exact test (*p* < 0.05) showing the direction of the observed value compared to the theoretical value for significant color and orthonasal sensory attributes of the Syrah wines evaluated by an expert panel (n = 15).

Treatment	Saturation	Purple Hue	Overall Aroma Intensity	Blueberry	Jammy	Meaty	Herbal	Acetaldehyde	Reduction
PD_Chap	> ^1,2^	>	<	>	<	<	<	>	<
*p*-value ^3^	***	***	n.s.	**	**	**	n.s.	n.s.	*
SubCap_Nat	>	>	>	<	<	>	<	<	>
*p*-value	*	***	*	n.s.	***	**	**	*	**
SubCap_Chap	>	>	>	<	<	>	>	<	>
*p*-value	n.s.	n.s.	n.s.	*	n.s.	*	n.s.	n.s.	n.s.
EM_Nat	<	<	>	>	>	<	>	>	<
*p*-value	***	***	n.s.	n.s.	**	*	*	n.s.	n.s.
EM_Chap	<	<	<	<	>	<	>	>	<
*p*-value	***	***	n.s.	n.s.	***	n.s.	n.s.	**	n.s.

^1^ Red coloration indicates statistical significance. ^2^ >: observed value was greater than predicted value; <: observed value was less than the predicted value; ^3^ n.s.: not significant; * *p* < 0.05; ** *p* < 0.01; *** *p* < 0.0001. PD: punch down; SubCap: submerged cap; EM: extended maceration; Nat: natural; Chap: chaptalized.

**Table 5:** Fisher’s exact test (*p* < 0.05) showing the direction of the observed value compared to the theoretical value for retronasal aroma and mouthfeel sensory attributes of the Syrah wines evaluated by an expert panel (n = 15).

Treatment	Black Fruit	Jammy	Dried Fruit	Meaty	Herbal	Baking Spice	Acetaldehyde	Reduction	Satin
PD_Chap	> ^1,2^	>	<	<	<	>	<	<	<
*p*-value ^3^	*	*	*	n.s.	*	n.s.	n.s.	n.s.	*
SubCap_Nat	<	<	<	>	<	<	<	>	>
*p*-value	n.s.	***	n.s.	**	n.s.	**	*	**	n.s.
SubCap_Chap	>	<	<	>	<	>	<	>	>
*p*-value	n.s.	n.s.	n.s.	n.s.	n.s.	n.s.	n.s.	*	n.s.
EM_Nat	<	>	>	<	>	<	>	<	<
*p*-value	n.s.	n.s.	*	*	*	n.s.	n.s.	**	n.s.
EM_Chap	<	>	>	<	>	>	>	<	>
*p*-value	n.s.	n.s.	*	*	n.s.	n.s.	**	n.s.	n.s.

^1^ Red coloration indicates statistical significance. ^2^ >: observed value was greater than predicted value; <: observed value was less than the predicted value; ^3^ n.s.: not statistically significant; * *p* < 0.05; ** *p* < 0.01; *** *p* < 0.0001. PD: punch down; SubCap: submerged cap; EM: extended maceration; Nat: natural; Chap: chaptalized.

## Data Availability

The data will be made available by the authors upon request.

## Supplementary Materials

The following supporting information can be downloaded at: https://www.mdpi.com/article/10.3390/molecules30081694/s1, Figure S1: Experimental winemaking design for Syrah wines made in the 2022 vintage. All treatments were established in triplicate. Sensory analysis occurred at 3 months of skin contact; Figure S2: Modified Pivot© Profile questionnaire for color assessment; Table S1: One-way analysis of variance (ANOVA) of the phenolic composition of Syrah wines at the time of sensory analysis. Values represent the mean of three replicates followed by the standard error of the mean; Table S2: Compounds analyzed using SPME and SBSE on the GC-MS, CAS numbers, manufacturers, and purity of Syrah wines; Table S3: Color, orthonasal aroma, taste, retronasal aroma, and mouthfeel attribute standard compositions for Syrah wines; Table S4: CATATIS analysis by each panelist (n = 15) showing agreement among the panel. Table S5: Similarity index to demonstrate the consensus among panelists (n = 15); Table S6: Comparison of replicates to indicate collective panelist (n = 15) repeatability.

## Abbreviations

The following abbreviations are used in this manuscript:

TDS	Temporal Dominance of Sensations
SPE	Solid-phase extraction
ACE	Accentuated cut edges
SPME	Solid-phase microextraction
PP	Pivot© Profile
DA	Descriptive analysis
CATA	Check-All-That-Apply
ANOVA	Analysis of variance
Chap	Chaptalized
Nat	Natural (not chaptalized)
SubCap	Submerged cap
PD	Punch down
EM	Extended maceration
MLV-3G	Malvidin-3-glucoside
TPP	Total polymeric pigments
CE	Catechin equivalents
PD_Nat	Punch down, natural
PD_Chap	Punch down, chaptalized
SPP	Small polymeric pigments
LPP	Large polymeric pigments
EM_Nat	Extended maceration, natural
EM_Chap	Extended maceration, chaptalized
CA	Correspondence analysis
SubCap_Chap	Submerged cap, chaptalized
SubCap_Nat	Submerged cap, natural
O	Orthonasal aroma
R	Retronasal aroma
RATA	Rate-All-That-Apply
SBSE	Stir-bar sorptive extraction
PROP	6-n-propylthiouracil
LSD	Least Significant Difference
