# Supplementary material for: The Chemical and Sensory Impact of Cap Management Techniques, Maceration Length, and Ethanol Level in Syrah Wines from the Central Coast of California
Source: Molecules. 2025 Apr 10;30(8):1694. doi: 10.3390/molecules30081694 (PMC12029964; doi:10.3390/molecules30081694)
Supplement: Supplementary file 1 [file molecules-30-01694-s001.zip › molecules-3560774-supplementary/Figure S1.pdf]

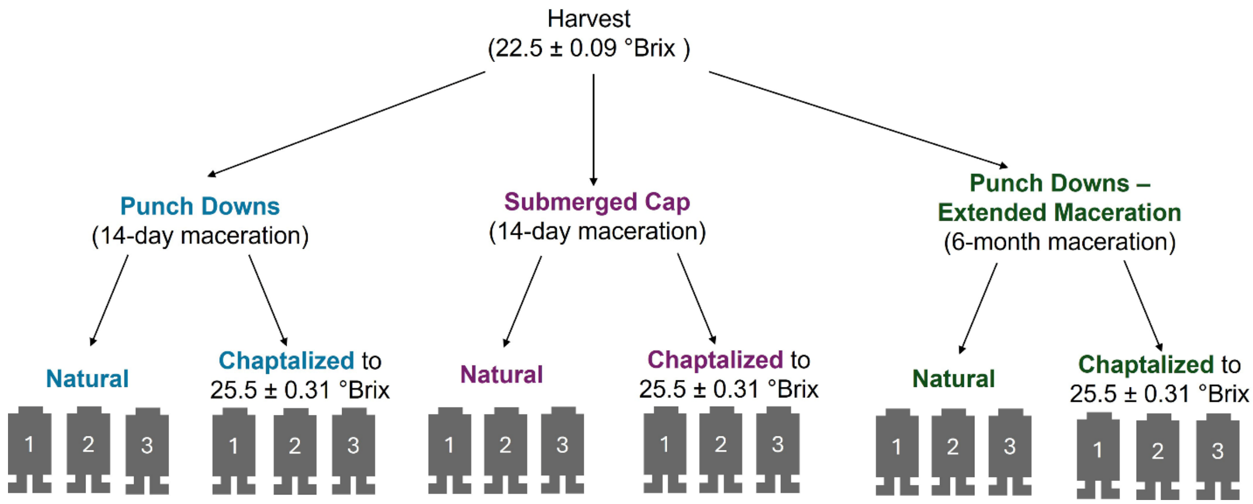


**Figure S1:** Experimental winemaking design for Syrah wines made in the 2022 vintage. All treatments were established in triplicate. Sensory analysis occurred at 3 months of skin contact
