# Supplementary material for: The Chemical and Sensory Impact of Cap Management Techniques, Maceration Length, and Ethanol Level in Syrah Wines from the Central Coast of California
Source: Molecules. 2025 Apr 10;30(8):1694. doi: 10.3390/molecules30081694 (PMC12029964; doi:10.3390/molecules30081694)
Supplement: Supplementary file 1 [file molecules-30-01694-s001.zip › molecules-3560774-supplementary/Figure S2.pdf]

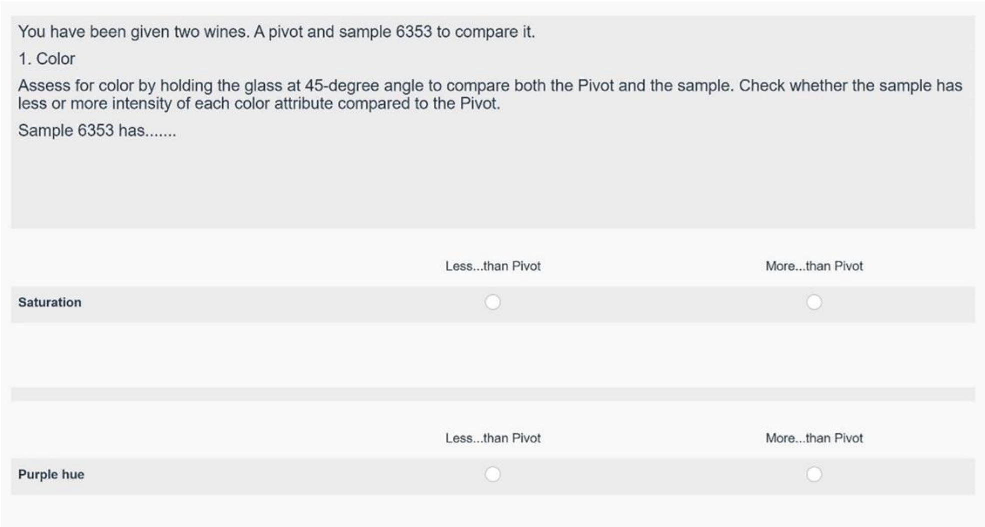

You have been given two wines. A pivot and sample 6353 to compare it.

1. Color

Assess for color by holding the glass at 45-degree angle to compare both the Pivot and the sample. Check whether the sample has less or more intensity of each color attribute compared to the Pivot.

Sample 6353 has.......

	Less...than Pivot	More...than Pivot
**Saturation**	○	○

	Less...than Pivot	More...than Pivot
**Purple hue**	○	○

Figure S2: Modified Pivot© Profile questionnaire for color assessment.
