# Supplementary material for: The Chemical and Sensory Impact of Cap Management Techniques, Maceration Length, and Ethanol Level in Syrah Wines from the Central Coast of California
Source: Molecules. 2025 Apr 10;30(8):1694. doi: 10.3390/molecules30081694 (PMC12029964; doi:10.3390/molecules30081694)
Supplement: Supplementary file 1 [file molecules-30-01694-s001.zip › molecules-3560774-supplementary/Table S1.pdf]

Table S1. One-way analysis of variance (ANOVA) of the phenolic composition of Syrah wines at the time of sensory analysis. Values represent the mean of three replicates followed by the standard error of the mean.

Treatment	*Anthocyanins (mg/L MLV-3G)*	*SPP*	*LPP*	*TPP*	*Tannins (mg/L CE)*	*Total Phenolics (mg/L CE)*
PD_Nat	747 ± 30.5 b[1]	1.81 ± 0.02 a	0.72 ± 0.04 a	2.53 ± 0.05 ab	216 ± 13.0 a	836 ± 40.2
PD_Chap	848 ± 26.7 a	1.91 ± 0.11 a	0.95 ± 0.06 a	2.87 ± 0.16 a	240 ± 9.87 a	851 ± 18.1
Sub_Nat	699 ± 19.7 b	1.85 ± 0.19 a	0.35 ± 0.17 bc	2.20 ± 0.36 b	112 ± 10.9 c	772 ± 30.8
Sub_Chap	731 ± 33.0 b	1.90 ± 0.15 a	0.42 ± 0.08 b	2.32 ± 0.23 ab	137 ± 12.0 bc	777 ± 24.2
EM_Nat	493 ± 26.6 c	1.19 ± 0.03 b	0.12 ± 0.03 c	1.31 ± 0.01 c	151 ± 10.5 b	747 ± 25.3
EM_Chap	544 ± 33.0 c	1.26 ± 0.01 b	0.21 ± 0.08 bc	1.47 ± 0.09 c	158 ± 16.9 b	731 ± 31.3
p-value[2]	***	**	**	**	***	n.s.

[1]*Different letters within the same column indicate a significant difference for Fisher's Least Significant Difference test ($p < 0.05$).*

[2] n.s.: not statistically significant; * $p < 0.05$; ** $p < 0.01$; *** $p < 0.0001$

MLV-3G: Malvidin-3-glucoside; SPP: Small polymeric pigments; LPP: Large polymeric pigments; TPP: Total polymeric pigments; CE: Catechin equivalents.

PD: Punch downs; SubCap: Submerged cap; EM; Extended maceration; Nat: Natural; Chap: Chaptalized.
