# Supplementary material for: The Chemical and Sensory Impact of Cap Management Techniques, Maceration Length, and Ethanol Level in Syrah Wines from the Central Coast of California
Source: Molecules. 2025 Apr 10;30(8):1694. doi: 10.3390/molecules30081694 (PMC12029964; doi:10.3390/molecules30081694)
Supplement: Supplementary file 1 [file molecules-30-01694-s001.zip › molecules-3560774-supplementary/Table S2.pdf]

Table S2: Compounds analyzed using SPME and SBSE on the GC-MS, CAS numbers, manufacturers, and purity for Syrah wines.

Compound	*CAS Number*	*Manufacturer*	*Purity*
Esters			
Ethyl isobutyrate	97-62-1	Alfa Aesar	98.0%
Ethyl butyrate	105-54-5	Alfa Aesar	99.0%
Ethyl propionate	105-37-3	TCI	99.0%
Isoamyl acetate	123-92-2	Acros Organics	99.0%
Hexyl acetate	142-92-7	Sigma Aldrich	98.5%
Ethyl hexanoate	123-66-0	TCI	99.0%
Ethyl heptanoate	106-30-9	Sigma Aldrich	99.0%
Ethyl octanoate	106-32-1	TCI	98.0%
Ethyl decanoate	110-38-3	TCI	98.0%
Diethyl succinate	123-25-1	Sigma Aldrich	99.5%
Ethyl hexadecanoate	56219-10-4	TCI	95.0%
Phenylethyl acetate	103-45-7	Sigma Aldrich	99.0%
Ethyl cinnamate	4192-77-2	TCI	99.0%
Ethyl lactate	97-64-3	Spectrum	98.0%
Nor-isoprenoids			
β-Damascenone	23726-91-2	Sigma Aldrich	90.0%
β-Ionone	14901-07-6	Sigma Aldrich	96.0%
Terpenes			
Citronellol	7540-51-4	Sigma Aldrich	95.0%
trans-Farnesol	4602-84-0	Sigma Aldrich	96.0%
Nerolidol	40716-66-3	Sigma Aldrich	85.0%
Alcohols			
1-Hexanol	111-27-3	Sigma Aldrich	99.0%
1-Octanol	111-87-5	Sigma Aldrich	99.0%
1-Nonanol	143-08-8	Sigma Aldrich	98.0%
Isoamyl alcohol	123-51-3	Sigma Aldrich	95.0%
Isobutanol	78-83-1	Fischer Chemical	95.0%
Phenylethyl alcohol	98-85-1	Acros Organics	98.0%
Aldehydes			
Benzaldehyde	10-52-7	Sigma Aldrich	99.0%
Sulfur Compounds			
Methionol	505-10-2	Sigma Aldrich	98.0%
Internal Standard			
2-Undecanone	112-12-9	Sigma Aldrich	98.0%
