# Supplementary material for: The Chemical and Sensory Impact of Cap Management Techniques, Maceration Length, and Ethanol Level in Syrah Wines from the Central Coast of California
Source: Molecules. 2025 Apr 10;30(8):1694. doi: 10.3390/molecules30081694 (PMC12029964; doi:10.3390/molecules30081694)
Supplement: Supplementary file 1 [file molecules-30-01694-s001.zip › molecules-3560774-supplementary/Table S3.pdf]

Table S3. Color, orthonasal aroma, taste, retronasal aroma, and mouthfeel attribute standard compositions for Syrah wines.

Attribute	*Standard composition*
	Color
Purple Hue	High: L* = 39.9, C* = 61.9, a* = 60.8, b* = 11.6[1]<br>Low: L* = 86.4, C* = 15.6, a* = 15.1, b* = 3.66[2]
Saturation	High: L* = 39.9, C* = 61.9, h = 10.8[1]<br>Low: L* = 86.4, C* = 15.6, h = 13.6[2]
	Orthonasal and Retronasal aromas[3]
Black fruit	60.0 g Blackberries (Driscoll's, Driscroll's Inc., Watsonville, California, USA), 56.0 g Dried prunes (First Street, Amerifoods Trading Co, Los Angeles, CA, USA), 24.5 g Blackberry Syrup (Torani, R. Torre & Company, San Leandro, CA, USA), 60.0 g marionberry jam (Dickinson's, The Dickinson Family, Inc., Oxnard, CA, USA)
Blueberry	53.9 g Blueberries (Private Selection, The Kroger Co., Cinncinati, OH, USA), 266 g Wild blueberry jam (Bonne Maman, Andros, France) heated for a 1 minute on High
Dried fruit	90.2 g chopped dried prunes (First Street, Amerifoods Trading Co, Los Angeles, CA, USA), and 51.4 g of seedless raisins (Kroger Co., Kroger Co., Cincinnati, OH, USA)
Jammy	159 g Four Fruit Preserves (Bonne Maman, Andros, France)
Black pepper	2.51 g Ground Black Pepper (First Street, Amerifoods Trading Co, Los Angeles, CA, USA)
Meaty	86.1 g 93% lean ground beef (First Street, Amerifoods Trading Co, Los Angeles, CA, USA) cooked on medium until browned
Herbal[4]	0.72 g mint (The Spice Hunter, The Spice Hunter, Inc., San Luis Obispo, CA, USA), 0.37 g sage (Simply Organic, Frontier Co-Op, Norway, IA, USA), 1 eucalyptus tea bag (Traditional Medicinals, Traditional Medicinals, 4515 Ross Road, Sebastopol, CA, USA)
Mineral	225 g decorative filler rocks (Ashland, MSPCI, Irving, TX, USA)
Baking spice	1 whole cinnamon stick (First Street, Amerifoods Trading Co., Los Angeles, CA, USA), 1 tsp ground cloves (Frontier Co-Op, Frontier Co-Op, Norway, IA, USA), 1 tsp fennel (McCormick Gourmet Collection, McCormick & Co., Inc., Hunt Valley, MD, USA) cooked on low for 15 minutes
Hot[4]	200 mL vodka (New Amsterdam, New Amsterdam Spirits Company, Modesto, CA, USA)
Acetaldehyde	Deliciosa Manzanilla Sherry Sanlucar de Barrameda Valdespino
Reduction	43.8 g boiled red cabbage (Ready Pac foods, Bonduelle, Irwindale, CA, USA), 104 g boiled cabbage water, and 21.0 g red cabbage raw (Ready Pac foods, Bonduelle, Irwindale, CA, USA)
	Taste
Acidity	1.32 g tartaric acid (LD Carlson Company, LD Carlson Company, Kent, OH, USA)
Bitter	1.35 g caffeine pure anhydrous power (Sigma Aldrich Sigma-Aldrich Co., St. Louis, MO, USA)
	Mouthfeel
Overall astringency[3]	1.34 g tannic acid (Sigma-Aldrich, Lot #MKBX4069V, Sigma-Aldrich Co., St. Louis, MO, USA)
Sand (high)	Sand (Ashland, MSPCI, Irving, TX, USA)
Fine emery (medium)	Nail file (Revlon, Revlon Inc., New York City, New York, USA)
Satin (low)	Satin ribbon (Ashland, MSPCI, Irving, TX, USA)

[1] *2021 Graciano research wine, CIE Lab Measurements*

[2] *2021 Graciano research and 50 mL of water, CIE Lab Measurements*

[3] *Prepared in 1000 mL of Franzia Merlot (Franzia, Franzia Vineyards, Ripon, CA, USA)*

[4]*Prepared in 750 mL of Franzia Merlot (Franzia, Franzia Vineyards, Ripon, CA, USA)*
