# Supplementary material for: The Chemical and Sensory Impact of Cap Management Techniques, Maceration Length, and Ethanol Level in Syrah Wines from the Central Coast of California
Source: Molecules. 2025 Apr 10;30(8):1694. doi: 10.3390/molecules30081694 (PMC12029964; doi:10.3390/molecules30081694)
Supplement: Supplementary file 1 [file molecules-30-01694-s001.zip › molecules-3560774-supplementary/Table S4.pdf]

**Table S4.** CATATIS analysis each panelist (n = 15) showing agreement among the panel.

| *Panelist Code* | *Agreement Score* |
|---|---|
| 030 | 0.900 |
| 111 | 0.870 |
| 140 | 0.850 |
| 180 | 0.860 |
| 287 | 0.850 |
| 551 | 0.910 |
| 569 | 0.890 |
| 640 | 0.790 |
| 698 | 0.900 |
| 705 | 0.870 |
| 787 | 0.860 |
| 811 | 0.870 |
| 876 | 0.880 |
| 930 | 0.880 |
| 937 | 0.880 |
