# Supplementary material for: The Chemical and Sensory Impact of Cap Management Techniques, Maceration Length, and Ethanol Level in Syrah Wines from the Central Coast of California
Source: Molecules. 2025 Apr 10;30(8):1694. doi: 10.3390/molecules30081694 (PMC12029964; doi:10.3390/molecules30081694)
Supplement: Supplementary file 1 [file molecules-30-01694-s001.zip › molecules-3560774-supplementary/Table S5.pdf]

Table S5. Similarity index to demonstrate the consensus among panelists (n = 15).

Panelist Code	030	111	140	180	287	551	569	640	698	705	787	811	876	930	937
030	1.00	0.770	0.740	0.700	0.700	0.800	0.780	0.730	0.810	0.770	0.750	0.770	0.800	0.800	0.760
111	0.770	1.00	0.690	0.700	0.740	0.720	0.800	0.700	0.770	0.800	0.740	0.710	0.730	0.750	0.730
140	0.740	0.690	1.00	0.710	0.710	0.760	0.740	0.690	0.730	0.720	0.700	0.730	0.740	0.710	0.690
180	0.700	0.700	0.710	1.00	0.750	0.850	0.710	0.600	0.780	0.740	0.730	0.730	0.750	0.720	0.790
287	0.700	0.740	0.710	0.750	1.00	0.790	0.730	0.600	0.770	0.710	0.710	0.720	0.740	0.710	0.760
551	0.800	0.720	0.760	0.850	0.790	1.00	0.760	0.620	0.790	0.730	0.790	0.810	0.800	0.790	0.800
569	0.780	0.800	0.740	0.710	0.730	0.760	1.00	0.720	0.770	0.780	0.740	0.750	0.760	0.790	0.750
640	0.730	0.700	0.690	0.600	0.600	0.620	0.720	1.00	0.700	0.700	0.650	0.650	0.710	0.670	0.640
698	0.810	0.770	0.730	0.780	0.770	0.790	0.770	0.700	1.00	0.760	0.760	0.760	0.800	0.770	0.820
705	0.770	0.800	0.720	0.740	0.710	0.730	0.780	0.700	0.760	1.00	0.720	0.750	0.710	0.730	0.760
787	0.750	0.740	0.690	0.730	0.710	0.790	0.740	0.650	0.760	0.720	1.00	0.730	0.760	0.750	0.760
811	0.770	0.710	0.730	0.730	0.720	0.810	0.750	0.650	0.760	0.750	0.730	1.00	0.720	0.760	0.770
876	0.800	0.730	0.740	0.750	0.740	0.800	0.760	0.710	0.800	0.710	0.760	0.720	1.00	0.730	0.720
930	0.800	0.750	0.710	0.720	0.710	0.790	0.790	0.670	0.770	0.730	0.750	0.760	0.730	1.00	0.750
937	0.760	0.730	0.690	0.790	0.760	0.800	0.750	0.640	0.820	0.760	0.760	0.770	0.720	0.750	1.00
