# Supplementary material for: The Chemical and Sensory Impact of Cap Management Techniques, Maceration Length, and Ethanol Level in Syrah Wines from the Central Coast of California
Source: Molecules. 2025 Apr 10;30(8):1694. doi: 10.3390/molecules30081694 (PMC12029964; doi:10.3390/molecules30081694)
Supplement: Supplementary file 1 [file molecules-30-01694-s001.zip › molecules-3560774-supplementary/Table S6.pdf]

Table S6: Comparison of replicates to indicate collective panelist (n = 15) repeatability.

Replicate	*1*	*2*	*3*
1	1.00	0.940	0.900
2	0.940	1.00	0.920
3	0.900	0.920	1.00
